# Effects of Gas Type, Oil, Salts and Detergent on Formation and Stability of Air and Carbon Dioxide Bubbles Produced by Using a Nanobubble Generator

**DOI:** 10.3390/nano13091496

**Published:** 2023-04-27

**Authors:** Kaiyu Zhou, Vincent Maugard, Wenming Zhang, Joe Zhou, Xuehua Zhang

**Affiliations:** 1 Department of Chemical and Materials Engineering, University of Alberta, Edmonton, AB T6G 1H9, Canada; kzhou2@ualberta.ca (K.Z.);; 2 Department of Civil and Environmental Engineering, University of Alberta, Edmonton, AB T6G 1H9, Canada; 3 Disruptive Separation Technology Ltd. (DSTL), Edmonton, AB T6X 1M5, Canada

**Keywords:** microbubbles, oil, detergent, gas type, inorganic salts

## Abstract

Recent developments in ultrafine bubble generation have opened up new possibilities for applications in various fields. Herein, we investigated how substances in water affect the size distribution and stability of microbubbles generated by a common nanobubble generator. By combining light scattering techniques with optical microscopy and high-speed imaging, we were able to track the evolution of microbubbles over time during and after bubble generation. Our results showed that air injection generated a higher number of microbubbles (<10 μm) than CO_2_ injection. Increasing detergent concentration led to a rapid increase in the number of microbubbles generated by both air and CO_2_ injection and the intensity signal detected by dynamic light scattering (DLS) slightly increased. This suggested that surface-active molecules may inhibit the growth and coalescence of bubbles. In contrast, we found that salts (NaCl and Na_2_CO_3_) in water did not significantly affect the number or size distribution of bubbles. Interestingly, the presence of oil in water increased the intensity signal and we observed that the bubbles were coated with an oil layer. This may contribute to the stability of bubbles. Overall, our study sheds light on the effects of common impurities on bubble generation and provides insights for analyzing dispersed bubbles in bulk.

## 1. Introduction

Ultrasmall bubbles have tremendous potential in a wide range of applications, such as agriculture, mining, medicine and food production. Although there is still controversy over the differentiation of micro/nanobubbles [1,2,3,4], it is becoming clear that bubbles with small sizes (<1–10 μm) possess desirable properties that set them apart from large bubbles (>2–5 mm). One of the most significant advantages of ultrasmall bubbles is their longer residence time in water due to smaller imposed buoyancy and larger surface area per unit volume which greatly enhance surface interaction and mass transfer to the bulk [5,6]. In this case, ultrasmall bubbles can significantly improve nutrient delivery to plants in agriculture, leading to higher crop yields. With regard to medicine, ultrasmall bubbles hold great potential for enhancing medical imaging and precise drug delivery through application of ultrasound waves. To achieve the fluffy texture for better taste, ultrasmall bubbles are also injected during food production, such as ice cream and chocolate. Recent studies demonstrate that these bubbles have promising potential as separation agents in various industries, such as oil recovery and mineral processing [7,8]. For example, microbubbles greatly improve heavy oil recovery from concentrated slurries [9] and enhance the extraction of organic waste and valuable minerals [10,11]. Moreover, microbubbles have been found to be effective in defouling contaminated surfaces [12,13]. Numerous methods have been developed for producing micro/nanobubbles on the surface of large substrates or particles in controlled systems, such as solvent exchange [14], (photo)catalytic reactions [15,16,17,18], electrolysis [19,20,21] and droplet reactions [22,23]. However, when generating micro/nanobubbles in large quantities, maintaining a large volume of water entirely free from impurities becomes an extremely challenging task with respect to studying bubble behavior in pure water. Therefore, a strategy for understanding and controlling the formation and stability of micro/nanobubbles is to investigate their behavior in the presence of an added substance of known entities.

The formation and stability of micro/nanobubbles can be impacted by pressure, gas type, oily substance, salts and detergents, among other factors. For instance, the solubility of gas is related to bubble size and stability, with oxygen bubbles having higher stability compared to air bubbles in the generation process due to rapid gas diffusion to the liquid occurring at a larger concentration gradient at the interface [24]. Additionally, the higher solubility of gas may inhibit gas diffusion, leading to relatively larger bubbles. Gas–water interfaces are also prone to adsorption of surface active materials, such as surfactants, which can enhance bubble stability up to the critical micelle concentration [25]. Moreover, the presence of salts may play a role in generating and stabilizing bubbles with different types of salts exhibiting ionic specificity [26]. Some salts decrease gas solubility while others may prevent bubble coalescence by activating the ion shielding effect [27,28]. Organic substances and gas bubbles both have hydrophobic surfaces, which contribute to an attractive interaction. A thin layer of heavy oil was found to form around the bubble surface after attachment, effectively preventing gas diffusion [28,29,30]. Similarly, the presence of organic substances can also contribute to the formation and stability of micro/nanobubbles produced from a nanobubble generator.

Several techniques requiring high energy input have been developed to produce a large number of micro/nanobubbles, such as electrolysis [31], hydrodynamic cavitation [9,32] and ultrasound [33,34]. The swirling-flow nanobubble generator is an efficient hydrodynamic cavitation method that can continuously produce enormous quantities of bulk nanobubbles. Although many previous studies characterize nanobubbles, microbubbles produced concurrently by a nanobubble generator have been largely neglected. These micron-sized bubbles may play a crucial role in the evolution of nanobubbles. Techniques such as high-speed imaging, DLS and focused beam reflectance measurement (FBRM) have been applied to study bulk bubbles. Among them, FBRM is particularly useful for analyzing the size and number distribution of small particles by analyzing the reflected laser signals [35,36,37,38,39], while DLS measures the scattering intensity from suspended objects ranging from a few nanometers to several micrometers.

In this study, we focused on formation and stability of micron-sized bubbles that were generated by turbulent shear under high pressure. In situ analysis was performed using FBRM to investigate the effects of gas type, salts, oil and surfactants on the size distribution and stability of microbubbles produced by using a common nanobubble generator that is commercially available. The DLS technique was used to study the change of the scattering intensity from objects in treated water by the nanobubble generator as a function of time. Our study shows that the number density and stability of air and CO_2_ microbubbles vary in a different way with the type of additives in water. Our findings may help identify conditions for producing more microbubbles.

## 2. Materials and Methods

### 2.1. Bubble Generation by Using a Nanobubble Generator

**Figure 1:**
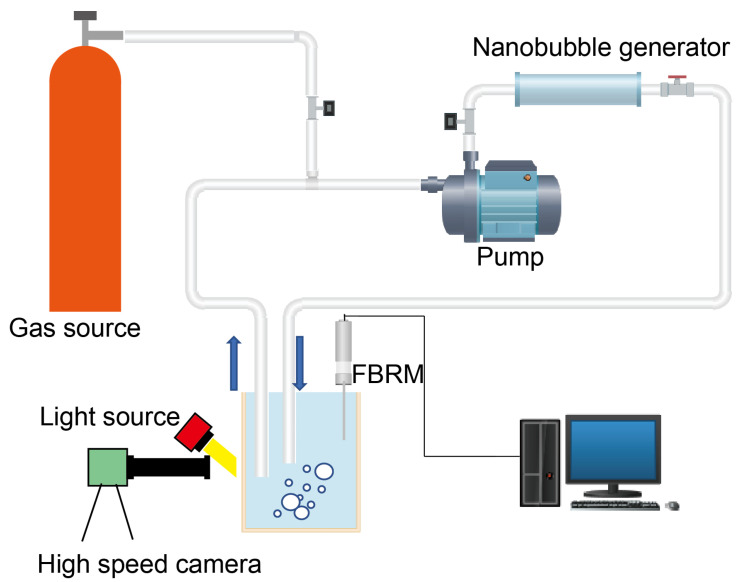
Sketch of nanobubble generator system installed with a high-speed camera for visualization of bubbles.

In our experiments, we used a commercial nanobubble generator from Newman Tech, which utilizes shear forces to create bubbles by pulling gas into the channel due to a velocity gradient. The setup included the nanobubble generator, a high-pressure pump and a gas source, as shown in Figure 1. The picture of the nanobubble generator system is displayed in Figure S1a. Initially, we prepared a 20 L volume of tap water at room temperature in an open tank. The composition of tap water is detailed in Table S1. Compressed air or CO_2_ and water was supplied to the high-pressure pump inlet, which was then connected to the nanobubble generator inlet.

To circulate the bubbly flow, we used the pump to push the water–gas mixture through the nanobubble generator. A valve installed at the exit of the nanobubble generator regulated the pressure at the outlet of the pump. The experimental conditions are summarized in Table 1. To optimize bubble generation, we increased the outlet pressure of the water–gas mixture to over 60 psi. We conducted a control measurement of bubbles generated at 58 psi. Additionally, we measured the gas flow rate at the inlet of the pump and recorded a minimum value of 0.4 L/min. An additional control test at 1 L/min was conducted to identify the effects of a higher gas flow rate.

Table 1 shows the substances added to the water, including detergent, salts (NaCl and Na_2_CO_3_) and soybean oil (Mukwano), before the nanobubble generator treatment. We maintained the outlet pressure and gas flow rate at 62 psi and 0.4 L/min, respectively. During the treatment process, the added substances quickly dissolved or dispersed in the water.

Surfactants have been widely applied to stabilize bubbles for a longer lifetime in the bulk. In this study, we add the hand detergent to the tap water being processed at concentrations from 2.5 to 20 ppm.

Depending on the type and concentration of salts, electrolytes in water have complex effects on liquid properties including viscosity, surface tension and solubility [40]. Specific anions influence the surface tension more than cations [41]. Thus, sodium salts with various anions are selected to learn the effect of electrolytes on bubble generation in our study. The sodium salts are added at a concentration of 50 ppm.

### 2.2. Visualization of Bubbles

As shown in Figure S1b, a transparent water tank was used to visualize the generated CO_2_ bubbles in the flow. The bubble generation was carried out at room temperature with a gas flow rate of 0.4 L/min and pressure of 62 psi. A high-speed camera (Basler) was positioned 6 cm away from the wall of the tank and was set perpendicular to the wall as presented in Figure S1b. To obtain a maximum magnification of 13×, we connected the camera to Navitar’s Zoom 6000 Lens System.

We recorded the videos at a resolution of 2040 × 1086 pixels and a frame rate of 165 fps. To enhance image quality at low exposure time, we used an intensified light source located next to the high-speed camera. The images captured have dimensions of 8 mm length and 4 mm width. However, the background of the videos appeared a bit blurry due to the light reflected by bubbles at the back of the focused plane. Those images were further analyzed via ImageJ software to obtain the size and number distribution of bubbles. To process the image, we first converted the RGB format to 8-bit, then applied a bandpass filter function using ImageJ to removed the background noise. Next, we used ilastik software to automatically filter any remaining background noise in the image and then imported the processed image back into ImageJ for threshold adjustment. Finally, we used the particle tracking function to measure the size and number of bubbles.

We separately added detergent and oil to the bulk at concentrations of 5 ppm and 6 ppm, respectively, to study the differences in bubble sizes. Optical microscopy was used to observe the bubbles in the presence of oil. For better image quality, a dye (Nile red) was dissolved in the vegetable oil. Several drops of water treated with a nanobubble generator were deposited on the glass slice.

### 2.3. Characterization of Bubbles

We used FBRM to measure the size and number distribution of microbubbles in the solution. FBRM is capable of detecting bubbles ranging from 1 to 1000 μm in size. As the number of bubbles larger than 10 μm was low, we used the number of bubbles smaller than 10 μm for comparison. During the 15-minute measurement, we recorded data every 2 s for each test.

We used DLS to analyze the bubbles generated in the solution. DLS measures the intensity of the scattered light, which is related to the Brownian motion of the dispersed tiny bubbles while large bubbles float up quickly. To investigate the stability of the bubbles, samples were taken from the water tank 10 min after bubble generation and tested immediately, followed by multiple times with a 5-minute interval between tests. Each test was performed for several minutes.

## 3. Results and Discussion

### 3.1. Visualization and Size Determination of Microbubbles

**Figure 2:**
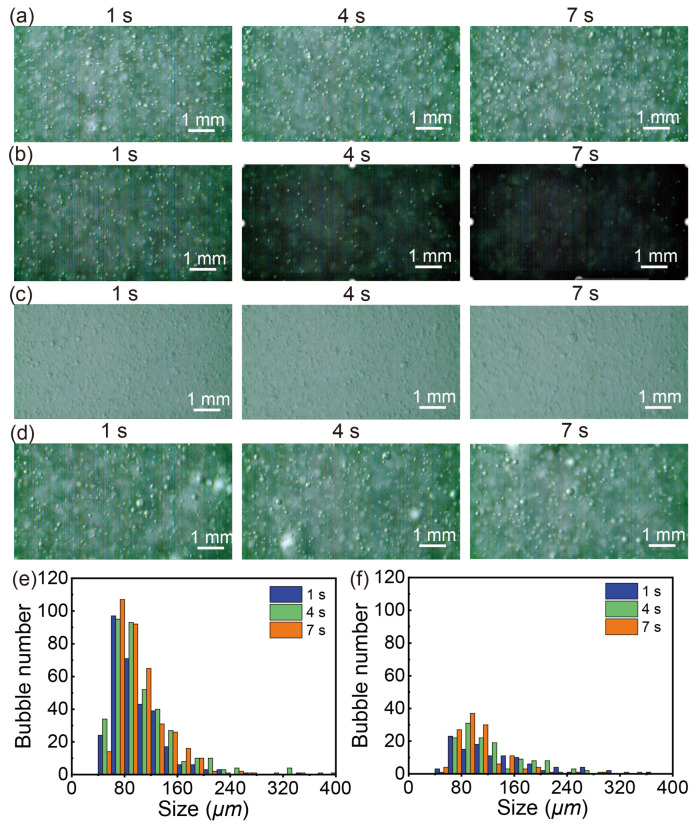
(**a**) Bubbles in tap water at 1, 4 and 7s with air flow rate of 0.4 L/min and pressure of 62 psi, (**b**) bubbles in tap water as the nanobubble generator shuts down, (**c**) bubbles in tap water with detergent addition at 1, 4 and 7 s with air flow rate of 0.4 L/min and pressure of 62 psi, (**d**) bubbles in tap water with oil addition at 1, 4 and 7s with air flow rate of 0.4 L/min and pressure of 62 psi, (**e**) the size and number distribution of bubbles as shown in (**c**), (**f**) the size and number distribution of bubbles as shown in (**d**).

Figure 2 illustrates the bubbles at 3-second intervals during the water treatment process using the nanobubble generator. As shown in Figure 2a,b, it can be observed that the microbubbles are evenly dispersed throughout the water with some larger bubbles. Interestingly, the number of visible bubbles quickly drops within 7 s after the operation of the nanobubble generator stops. Concurrently, the background gradually darkens with time as the reflected and scattered light from bubbles becomes weaker with the decrease in the bubble number density.

Figure 2c presents the bubbles generated during the water treatment process with the addition of detergent. The addition of detergent results in a homogeneous and opaque background. Distinct from that in Figure 2a where the generated bubbles were easily distinguishable from the background, the bubbles are difficult to distinguish from the surrounding environment in Figure 2c due to the high number of bubbles produced. The higher density of these bubbles scatter light strongly. Bubbles generated with the addition of oil are shown in Figure 2d. The background looks similar to that in water shown in Figure 2a. However, the number density of bubbles is much lower and the size of bubbles is comparatively larger.

**Figure 3:**
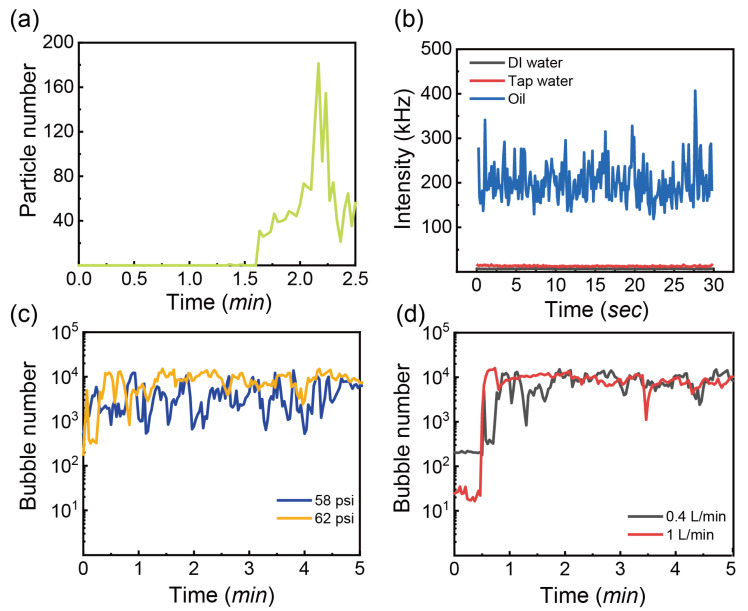
(**a**) Particle number (<10 μm) as a function of time without treatment, (**b**) Intensity signal of Milli-Q water, tap water and oil as a function of time at room temperature, (**c**) CO_2_ bubble number (<10 μm) as a function of time at the gas flow rate of 0.4 L/min and pressure of 58 and 62 psi, (**d**) CO_2_ bubble number (<10 μm) as a function of time at the gas flow rate of 0.4 and 1 L/min and pressure of 62 psi.

The measurements by FBRM were performed for untreated water to obtain the baseline signals. The resulting data displayed in Figure 3a show that the number of particles of size 1–10 μm was zero initially and increased with time. The maximal count peaked at approximately 180. The recorded count in untreated water could be related to a small number of particles in tap water. The count of 180 is set as the baseline signal.

Figure 3b shows the scattering intensity in water measured via DLS as a function of time before treatment. It is evident that both tap water and Milli-Q water scattered the light weakly with the signal intensity below 25 KHz (i.e., kilocounts per second), with a slightly higher signal intensity from tap water. This could be attributed to impurities in tap water, such as nanoplastics and minerals [42,43]. As comparison, the signal intensity from soybean oil was much higher than in water, fluctuating from 125 to 425 kHz. The stronger scattering from the oil could be attributed to a higher refractive index of oil than water [44]. Additionally, there are always long chain lipids in natural oil that scatter the light strongly.

We note that FBRM is unable to differentiate bubbles and impurities and the impurities in water can be counted as well. In the measurements via FBRM, a focused laser beam from the probe spins at a high speed. Two successive signals of back scattered light can be detected as the laser beam intersects with two opposite edges of an object [45]. The measured length called the chord length is the product of the velocity of the laser and the time of the interval between two signals.

Lack of chemical identification is also a limitation for the DLS technique that analyzes the scattering of light by shining a laser beam at the liquid medium. The scattering intensity is influenced by other significant factors, such as sample concentration, shape of particles and aggregation of particles [46,47,48,49]. Quantitatively, the hydrodynamic radius of the particle can be determined with the Stokes–Einstein equation:

(1) $$D_{t}=\frac{k_{B}T}{6\pi \eta R_{H}}$$

Here $D_{t}$ is translational diffusion coefficient, $k_{B}$ is Boltzmann constant, *T* is temperature, η is absolute viscosity of the solvent and $R_{H}$ is the hydrodynamic radius. $D_{t}$ can be calculated through the autocorrelation function which is also related to the refractive index of the solvent and the settings of the instrument. Similar to FBRM, the DLS technique cannot distinguish the bubbles from droplets and solid particles in the fluid for all our following measurements.

### 3.2. Dependence of Bubble Sizes on Gas Flow Rate, Pressure of the Generator, Gas Type and Temperature

The operation conditions of the nanobubble generator are important for the size and number of bubbles. Figure 3c,d shows the time-dependent number of CO_2_ bubbles (1–10 μm) at various inlet pressure and gas flow rates. As the gas flow rate is 0.4 L/min, the bubble number has large fluctuations from 100 to 20,000 at the outlet pressure of the pump at 58 and 62 psi. There are a larger number of bubbles at the inlet pressure of 62 psi, which indicates that at a higher pressure of the pump more bubbles are generated.

Figure 3d shows that increasing the gas flow rate to 1 L/min makes little difference to the time-dependent bubble number at 62 psi. According to the working principle of the nanobubble generator, the shearing energy breaks the gas into small bubbles. For a large gas flow rate, the efficiency of mechanical shearing in decreasing the mean bubble diameter is weakened owing to the energy dissipation to a larger gas volume [50,51]. In addition, at the higher gas flow rate, bubbles coalesce more at a higher gas fraction [9]. However, the high pressure gradient in the chamber can force the deformation of bubbles and in turn contributes to the breakup of large bubbles [52]. Furthermore, the improved solubility of CO_2_ at the high pressure may influence the bubble generation. The combination of inlet pressure of 62 psi and gas flow rate of 0.4 L/min is applied for the following studies.

**Figure 4:**
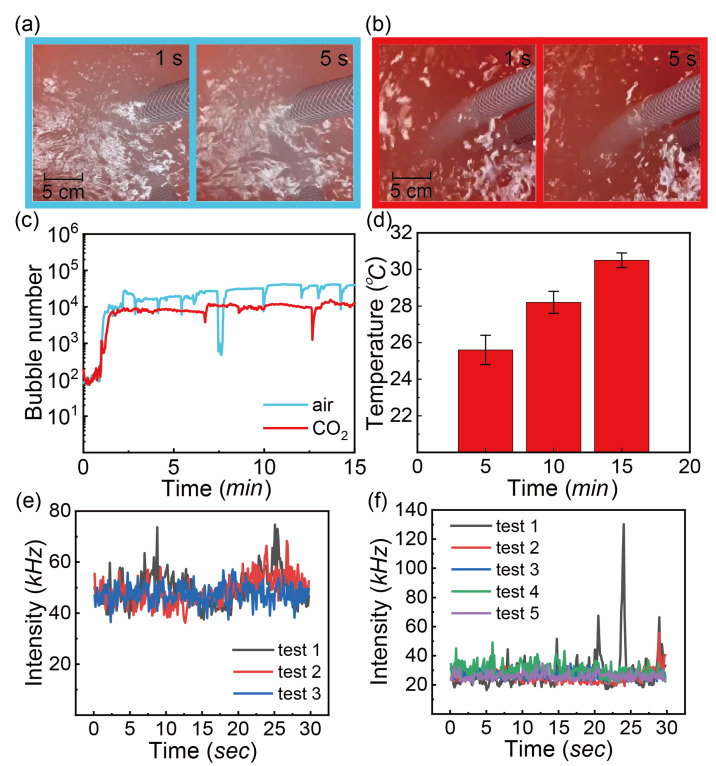
The observation of water with the treatment of nanobubble generator at gas flow rate of 0.4 L/min and pressure of 62 psi: (**a**) air injection; (**b**) CO_2_ injection; (**c**) Bubble number (<10 μm) as a function of time with injection of air and CO_2_; (**d**) the real-time temperature of tap water with CO_2_ injection at 5, 10 and 15 min; the intensity of scattered laser as a function of time: (**e**) air injection; (**f**) CO_2_ injection; test 1 starts from 0 min and the gap of each test is 5 min.

Bubbles are generated from air and CO_2_ injection. The photos in Figure 4a,b show that bubbly water from air injection is more turbid than that from CO_2_ injection. Figure 4c presents the microbubble number measured by FBRM. The count of bubbles from air injection is consistently higher than that from CO_2_ injection, indicating that at the same gas flow rate, more bubbles form from air than from CO_2_ injection. The difference between the air and $CO_{2}$ injection from FBRM measurements is consistent with the observation from the photos of the samples shown in Figure 4a,b.

In the process of CO_2_ bubble generation, the temperature of water was measured at 5, 10 and 15 min as shown in Figure 4d. The temperature climbed from 25 to 31 °C within 10 min. It can be inferred that the temperatures of water being treated under other conditions share the similar temperature trend because the temperature change is caused by heat from the nanobubble generator system. The working condition of the nanobubble generator system is consistent in the following experiments. However, temperature is an important factor influencing the solubility of CO_2_. It can be seen that the bubble number is relatively constant at 5, 10 and 15 min, as shown in Figure 4c.

The stability of air and CO_2_ bubbles was compared, based on the intensity in the light scattering from DLS measurements. Figure 4e,f show the scattering intensity as a function of time with air and CO_2_ injection as measured via DLS. The measurement was performed 10 min after operation of the nanobubble generator and the subsequent measurements were at 5 min intervals. The scattering intensity from either air or CO_2_ injection remains the same with time. However the scattering intensity of the approximate 50 kHz from air-treated water is consistently higher than that from CO_2_-treated water.

Compared to the baseline of water without treatment of 23 kHz, the stronger scattering from the treated water may indicate the presence of stable entities, perhaps including very tiny bubbles or impurities. The higher intensity of air-treated water might attribute to more stable entities. It is well known that CO_2_ has a higher solubility than air in water; therefore, a higher concentration of CO_2_ is needed to reach the same level of oversaturation from air injection. Meanwhile, CO_2_ bubbles are unstable, possibly due to the ionic strength in carbonated water. The dissolved CO_2_ molecules react with water and decrease the pH of the solution. Hamamoto et al. pointed out the repulsive interaction is weakened because the surface charge decreases with decreasing pH [53]. In addition, the surface potential of CO_2_ bubbles may be higher than air bubbles due to the lower pH from dissociation of the carbon acid group [54]. Hence, CO_2_ bubbles might more readily collide and coalesce to form large and buoyant bubbles. Another reason is the higher solubility of CO_2_ that might retard the gas diffusion rate out of any generated small bubbles, thereby giving rise to larger sizes of small bubbles than air.

### 3.3. Effects of Additives on Formation and Stability of Bubbles

In this section, water will contain additives of salts, oil or detergent. The concentration of each additive is varied at several levels, but all within the range of milligrams per liter (ppm). The counts of bubbles measured via FBRM and the stability of bubbles after the treatment as indicated by the scattering intensity from DLS measurements will be presented after the water is treated by the nanobubble generator with both air and CO_2_ as the gas supply.

#### 3.3.1. Salts

The aquatic environment in the industry is rich in many kinds of salts that may impact bubble formation and stability. We investigated the effect of NaCl and Na_2_CO_3_ on bubble generation in the water, with a concentration of 50 ppm for both sodium salts.

**Figure 5:**
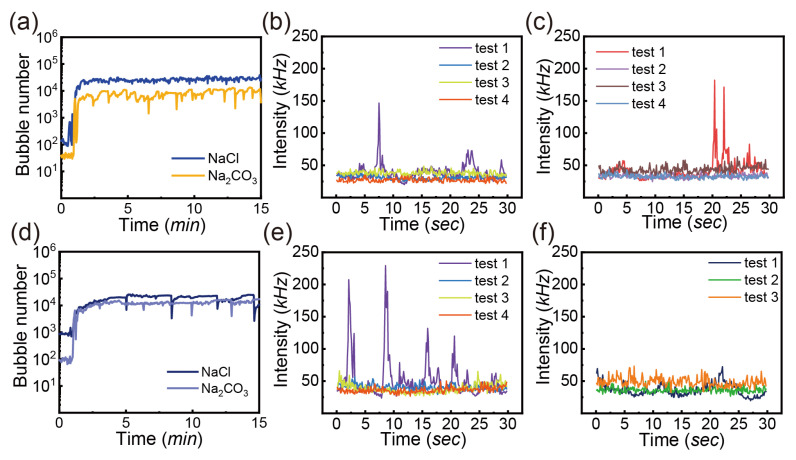
Bubble number (<10 μm) as a function of time with addition of sodium salts at concentration of 50 ppm at gas flow rate of 0.4 L/min and pressure of 62 psi: (**a**) air injection, (**d**) CO_2_ injection; the intensity of scattered laser in the air-injected sample as a function of time at different tests: (**b**) NaCl addition, (**c**) Na_2_CO_3_ addition; the intensity of scattered laser in the CO_2_-injected sample as a function of time at different tests: (**e**) NaCl addition, (**f**) Na_2_CO_3_ addition.

For air bubbles shown in Figure 5a–c, the bubble number in the NaCl solution is relatively constant over time, on the same order of magnitude as the bubbles in water without additives (Figure 4c). However, the bubble number in the Na_2_CO_3_ solution is consistently lower than that in the NaCl solution. Figure 5b,c shows the scattering intensity from NaCl and Na_2_CO_3_ solutions. Apparently, the scattering intensity is relatively constant (25–50 kHz), slightly weaker than that from water shown in Figure 4f. The scattering intensity does not support that the salts may stabilize small air bubbles.

For CO_2_ bubbles shown in Figure 5d–f, the count of bubbles with a size of <10 μm is slightly higher with addition of NaCl than that with addition of Na_2_CO_3_. Interestingly, the addition of two salts leads to a gentle improvement in the number of bubbles by >10,000 in the water. Figure 5e shows strong scattering peaks in the first test (i.e., 10 min after the generator operation) in NaCl solution, indicating the presence of large dispersed substances, possibly evolved bubbles and suspended impurities. The intensity is higher than that from the air-treated solution of NaCl. Figure 5f presents similar intensity from the CO_2_-treated solution of Na_2_CO_3_ as NaCl.

The decrease in the air bubble count after adding the salts in our results would be contradictory to the previous report that NaCl and Na_2_CO_3_ may prevent bubble coalescence [55,56]. According to ion-specificity relying on the combination of cation and anion, NaCl is regarded as a coalescence inhibitor with a transitional concentration of 0.078 M [57]. Simultaneously, the surface tension is found to have a positive linear relationship with NaCl concentration [58]. In our study, NaCl of 50 ppm is equal to $8.6\times 10^{-4}$ M which is smaller than 0.078 M. The low salt concentration and increased surface tension might contribute to the unstable bubbles. Alexandre et al. also showed that both NaCl and Na_2_CO_3_ increase the surface tension [40]. Moreover, the presence of Na_2_CO_3_ can form the mixed electrolyte solution with reaction of hydroxide and water, resulting in the alteration of chemical nature [58]. In this case, the effect on the surface tension may play a major role in the stability of bubbles.

For CO_2_ injection, adding both sodium salts slightly increases the number of CO_2_ bubbles. It is well known that the gas solubility is sensitive to the dissolved salts. The bubble coalescence might be hindered by reduced dissolved CO_2_ which influences the bubble interaction via the hydrophobic attraction [58]. Moreover, the reaction of hydroxide with water can form the mixed electrolyte solutions. Although the classical DLVO theory predicts that increasing salt concentrations generally increases the probability of coagulation or coalescence of colloids, further study is necessary for a better understanding in our cases.

#### 3.3.2. Oil

**Figure 6:**
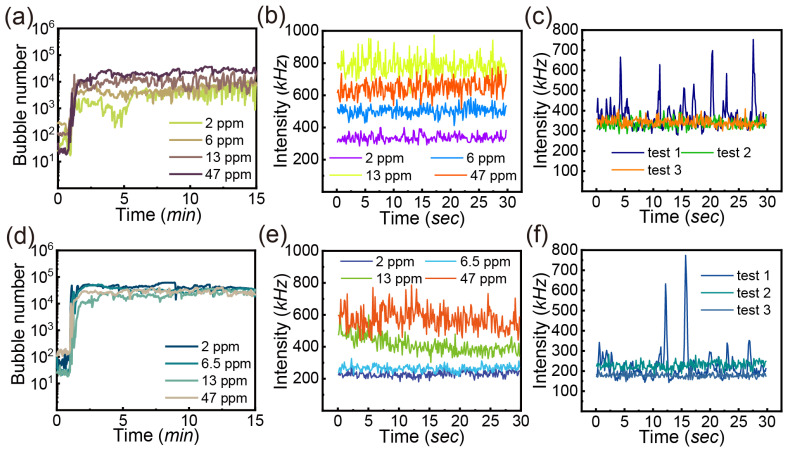
Bubble number (<10 μm) as a function of time with oil addition at gas flow rate of 0.4 L/min and pressure of 62 psi: (**a**) air injection with oil concentration of 2, 6, 13, 47 ppm, (**d**) CO_2_ injection with oil concentration of 2, 6.5, 13, 47 ppm; the intensity of scattered laser at test 2 as a function of time: (**b**) air injection with oil concentration of 2, 6, 13 and 47 ppm and (**e**) CO_2_ injection with oil concentration of 2, 6.5, 13, 47 ppm; the intensity of scattered laser at different tests as a function of time: (**c**) air injection with oil concentration of 2 ppm and (**f**) CO_2_ injection with oil concentration of 2 ppm.

With the addition of oil into water, we can compare the differences in air and CO_2_ injection. As shown in Figure 6a, the presence of oil in water leads to fluctuations in the number of bubbles (<10 μm). Figure 6b shows that the scattering intensity increases sharply with the increased oil concentration from 2, 6, 13 and 47 ppm. The scattering intensity of various tests with oil concentration of 2 ppm is quite consistent with significant fluctuations in Figure 6c.

The high scattering intensity with oil addition might be attributed to the higher refractive index of oil. The dispersed oil drops by shearing can lead to increased intensity as well. With increasing oil concentration, the number of oil drops might increase as well which leads to an increase in the intensity at a higher oil concentration.

As for CO_2_ injection, the bubble number as a function of time and oil concentration is shown in Figure 6d. Conversely, adding oil contributes to an increased number of bubbles from CO_2_ different from that from air, but the effect from increasing the concentration of oil is not obvious. Figure 6e demonstrates the scattering intensity of test 2 at various concentrations as a function of time. The magnitude of intensity increases with increasing oil concentration. In comparison, the intensity is lower with CO_2_ than that with air at the same oil concentration. In Figure 6f, at the oil concentration of 2 ppm, the scattering intensity has great fluctuation for all tests ranging from 150 to 350 kHz without counting the significant peaks. The acidic solution from dissolved CO_2_ in water can destabilize the oil drops in the bulk [59].

Bubbles are widely applied to separate organic substances due to strong hydrophobic interaction [9,60]. The interaction between bubbles and oil is dependent on the oil viscosity and temperature [61]. According to the two-stage model, the presence of tiny bubbles can bridge the oil covered bubbles and form larger aggregates [62]. This might decrease the bubble number as the flotation of large aggregates. Another mechanism is the engulfment of oil on bubbles, driven by the interfacial energy minimization. The oil layer acts as a shell to enhance the stability of oily bubbles [63].

**Figure 7:**
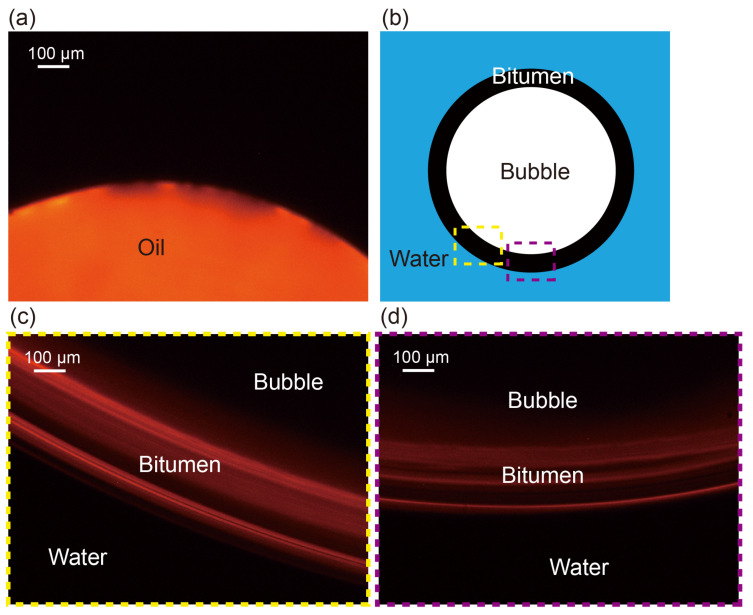
(**a**) Microscopic observation of dyed oil drop on the substrate, (**b**) sketch of oil-coated bubble; oil layer at two positions of the bubble are shown in (**c**,**d**).

To gain an insight into the effects of oil, the optical microscopy was used to visualize the interaction between bubble and oil surface. Figure 7 displays the dyed oil drop on the glass substrate. The water containing oil treated by a nanobubble generator was dropped between two slices along with a trapped air bubble. The red edge around the air bubble was captured under the red background. The red boundary clearly shows that the oil in the water forms a layer at the bubble surface. This phenomenon is in good agreement with the findings in the literature [63,64].

At room temperature, the surface tension of water ($\gamma_{W}$) and oil ($\gamma_{O}$) are 72.8 mN/m and 30 mN/m, respectively [65]. The interfacial tension between oil and water can be estimated through Fowkes’ equation:

(2) $$\gamma_{O/W}=\gamma_{O}+\gamma_{W}-2\sqrt{\gamma_{O}^{vdW}*\gamma_{W}^{vdW}}$$

$\gamma_{O/W}$ is 51.7 mN/m by calculation. Obviously, oil drops are more stabilized than air bubbles in the water. At the same time, it can be seen that a lower interfacial energy is obtained when bubbles are coated with oil. Basically, the free energy change for a single bubble engulfed by an oil drop is given by:

(3) $$\Delta G=\gamma_{O/W}\cdot A_{1}-\left(\gamma_{W}\cdot A_{2}+\gamma_{O/W}\cdot A_{3}\right)$$

Here $A_{1}$ is the area of the oil–water interface of oil drop, $A_{2}$ is the area of the air–water interface of bubble and $A_{3}$ is the area of the oil–water interface of oil coating. As the oil coating forms a thin layer on a bubble, the difference in $A_{1}$ and $A_{2}$ is negligible. In this case, we can obtain that the surface energy change is negative, which is a favorable process with respect to energy use.

#### 3.3.3. Detergent

**Figure 8:**
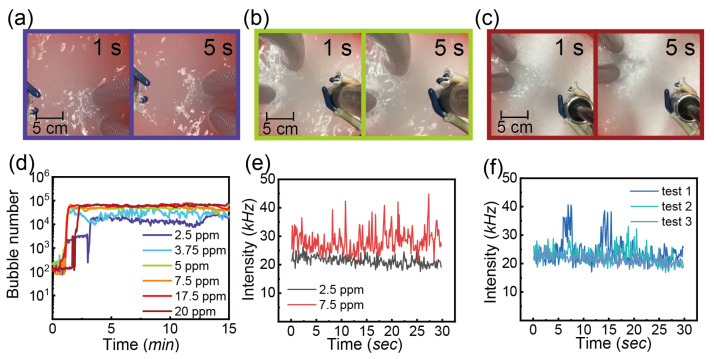
Water with the treatment of nanobubble generator. The air flow rate of 0.4 L/min and pressure of 62 psi. The concentrations of the detergent are: (**a**) 2.5 ppm, (**b**) 5 ppm and (**c**) 20 ppm, (**d**) Bubble number (<10 μm) as a function of time with injection of air and detergent concentration of 2.5, 3.75, 5, 7.5, 17.5 and 20 ppm, (**e**) the intensity of scattered laser of test 2 as a function of time with detergent concentration of 2.5 and 7.5 ppm, (**f**) the intensity of scattered laser as a function of time with detergent concentration of 2.5 ppm; test 1 starts at 0 min and the gap of each test is 5 min.

The last additive is a commercial detergent at different concentrations. As seen in Figure 8a–c, the foam layer floated at the top of the water and the thickness of foam increases with the detergent concentration from 2.5 ppm to 20 ppm. Figure 8d presents the bubble number (<10 μm) as a function of time with various detergent concentrations. At the lowest concentration of 2.5 ppm, there is no significant enhancement in bubble generation compared to the air bubble number in water, as shown in Figure 8c. However, the bubble number increases rapidly with an increase in the detergent concentration to 5 ppm. No further increase in the bubble number was observed by further increasing the detergent concentration.

Figure 8e shows the scattering intensity as a function of time with concentrations of 2.5 and 7.5 ppm at test 2 because the intensity signal is more stable than test 1 as mentioned earlier. It is obvious that the intensity increases slightly on increasing the detergent concentration. Moreover, the fluctuation of intensity was higher at 7.5 ppm. Similar to the previous finding, test 1 in Figure 8f shows larger peaks and the intensity of test 2 and test 3 is stabilized between 20 and 30 kHz.

**Figure 9:**
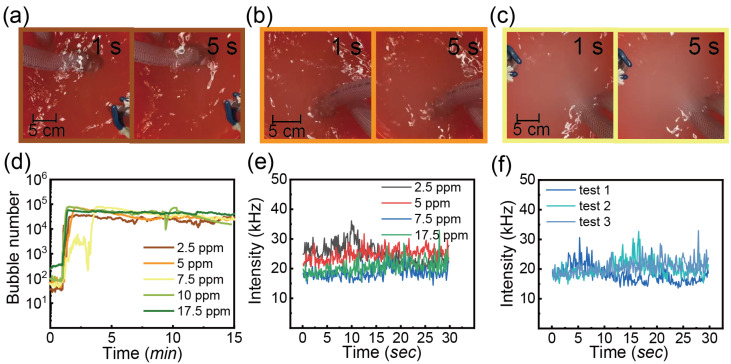
The observation of water with the treatment of nanobubble generator at CO_2_ flow rate of 0.4 L/min and pressure of 62 psi; the concentrations are: (**a**) 2.5, (**b**) 5 and (**c**) 7.5 ppm, (**d**) Bubble number (<10 μm) as a function of time with injection of CO_2_ and detergent concentration of 2.5, 5, 7.5, 17.5 ppm, (**e**) the intensity of scattered laser at test 2 as a function of time with detergent concentration of 2.5, 5, 7.5 and 17.5 ppm, (**f**) the intensity of scattered laser as a function of time with detergent concentration of 2.5 ppm; test 1 starts at 0 min and the gap of each test is 5 min.

For CO_2_ injection, the treated water with detergent concentration of 2.5, 5 and 7.5 ppm was displayed in Figure 9a–c. Different from the water with air injection, the water is quite transparent with the concentration of 2 and 2.5 ppm, but becomes a bit milky as more detergent was added in Figure 9c.

It is interesting to note the addition of detergent enhances the bubble generation as shown in Figure 9, even though the treated water looks transparent. The increase in the detergent concentration did not lead to a great increase in bubble production.

Figure 9e presents the intensity signal of test 2 of water with CO_2_ injection and the detergent concentration from 2.5 ppm to 17.5 ppm. The intensity signal overlaps without showing the obvious distinction caused by the concentrations. At the concentration of 2.5 ppm, the intensity exhibits consistency from test 1 to test 3 which means the tiny entities are stable within 15 min in Figure 9f. By contrast, the intensity signal with the detergent concentration of 2.5 ppm shows similarity for air and CO_2_ injection.

It is reported that the bubble size can decrease to 100 μm with the concentration of surfactant (sodium dodecyl sulfate), increasing to the critical point but with no further decrease in the size at higher concentrations [25,66]. The composition is complex in the commercial detergent. The typical formulation for hand wash consists of several key ingredients, including surfactants, humectants, preservatives, fragrances, colorants and water. The exact formulation and concentration of these ingredients may vary depending on the brand and type of hand wash. A surfactant in the composition of the hand wash used in our experiments is colateric COAB (chemical name of is 1-Propanaminium, 2-hydroxy-N, N-dimethyl-N-[3-[(1-oxyooctyl)amino]propyl]-3-sulfo-,inner salt), a high foaming amphoteric surfactant that builds the viscosity and stability of the foam [67,68]. Other compositions, such as the moisturizing agent PEG-75 Lanolin (ethoxylated Lanolin) and critic acid may all help the formation and stability of bubbles [69]. Our results imply that even a small amount of detergent is sufficient to increase the number of bubbles and increase the scattering intensity of water treated via the NB generator.

## 4. Conclusions

This study presents the effects of added substances on the formation and stability of air and CO_2_ microbubbles generated from the shearing process in a nanobubble generator. Air injection produces a larger number of microbubbles. Addition of detergent has a pronounced impact on the number of microbubbles. The presence of salts and oil has a negligible effect on the number of air bubbles. Conversely, an increase in number of CO_2_ microbubbles is obtained with the addition of salts and oil. To gain a more comprehensive understanding of the formation and stability of bubbles with the sizes below the detection limit of our techniques, more powerful techniques will be required for fast and precise characterization of bubbles in future studies. Overall, analyzing and understanding bubble formation affected by chemicals gives an insight into controlling number and size of bubbles produced by using a nanobubble generator. The addition of minute concentrations of specific bulk may improve the number of bubbles produced from the nanobubble generator and benefit the applications with slow processes with respect to interacting with bubbles.

## Figures and Tables

**Table 1:** Experimental conditions for micro/nanobubble generation in the tap water with chemicals. *P*: inlet pressure of mixture; $Q_{g}$: gas flow rate; *T*: water temperature.

Gas Type	Air	CO_2_
**Operating conditions**		
*P* (psi)	62	58, 62
$Q_{g}$ (L/min)	0.4	0.4, 1
*T* (°C)	20	20
**Chemical addition @ 62 psi, 20 °C and $Q_{g}$ of 0.4 L/min**		
Hand soap (ppm)	2.5, 3.75, 5,7.5, 17.5, 20	2.5, 5, 7.5,10, 17.5
NaCl (ppm)	50	50
Na_2_CO_3_ (ppm)	50	50
Soybean oil (ppm)	1, 2, 6, 13, 47	2, 6.5, 13, 47

## Data Availability

The data presented in this study are available on request from the corresponding author. The data are not publicly available due to privacy.

## Supplementary Materials

The following supporting information can be downloaded at: https://www.mdpi.com/article/10.3390/nano13091496/s1, Figure S1: Pictures of (a) nanobubble generator system, and (b) high-speed camera setup for visualization of bubbles generated in the water tank; Table S1: Compositions and physicochemical properties of substances. References [70,71] were cited in the supplementary materials.
