# Supplementary material for: Effects of Gas Type, Oil, Salts and Detergent on Formation and Stability of Air and Carbon Dioxide Bubbles Produced by Using a Nanobubble Generator
Source: Nanomaterials (Basel). 2023 Apr 27;13(9):1496. doi: 10.3390/nano13091496 (PMC10180106; doi:10.3390/nano13091496)
Supplement: Supplementary file 1 [file nanomaterials-13-01496-s001.zip › nanomaterials-2352953-supplementary.pdf]

Supplementary Materials

Effects of Gas Type, Oil, Salts and Detergent on Formation and Stability of Air and Carbon Dioxide Bubbles Produced by Using a Nanobubble Generator

Kaiyu Zhou [1], Vincent Maugard [1], Wenming Zhang [2], Joe Zhou [3], Xuehua Zhang [1,*]

[1] Department of Chemical and Materials Engineering, University of Alberta, Alberta T6G 1H9, Canada
[2] Department of Civil and Environmental Engineering, University of Alberta, Alberta T6G 2W2, Canada
[3] Disruptive Separation Technology Ltd.(DSTL), Edmonton, Alberta T6X 1M5, Canada
* Correspondence: xuehua.zhang@ualberta.ca

Table S1. Compositions and physicochemical properties of substances [1-2].

Detergent (Decon Labs. Inc)		Tap water	
**Composition**	**% in product**	**Parameter**	**Value**
Water	60-65	pH	7.8
LES-ME 27	19-23	Turbidity (NTU)	0.05
Colateric COAB	Trace	Total Organic Carbon (mg/L C)	1.6
PEG-75 Lanolin	Trace	Conductivity (μS/cm)	382
Citric Acid	Trace	Alkalinity Total (mg $CaCO_3$/L)	121
		Total Chlorine (mg/L)	2.06

(a) **(b)**

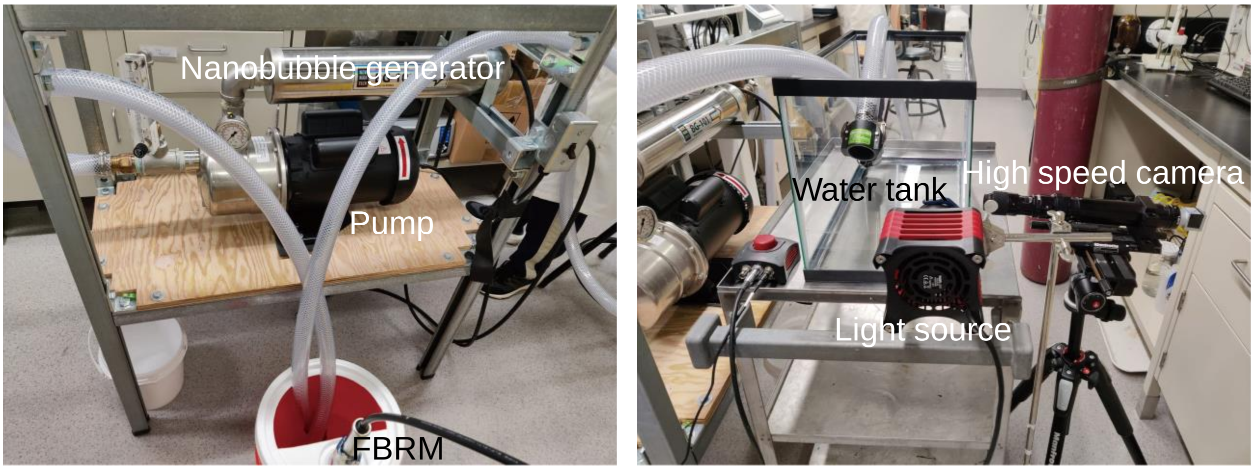

Figure S1. Pictures of (a) nanobubble generator system, and (b) high-speed camera setup for visualization of bubbles generated in the water tank.

References

1. https://deconlabs.com/products/skin-care-bacdown-handsoap/
2. https://www.epcor.com/products-services/water/water-quality/Pages/water-quality-reports-edmonton.aspx#/waterworks_
